# Multidomain Morbidity at 7–8 Years After Very Preterm Birth: Associations with Perinatal and Neonatal Factors

**DOI:** 10.3390/children13101333

**Published:** 2026-09-30

**Authors:** Ercan Yılmaz, Nezihe Koker Ozer, Hatice Turgut, Erdem Topal, Ramazan Ozdemir, Recep Gunakın, Mehmet Aslan

**Affiliations:** 1 Department of Pediatric Allergy and Immunology, Faculty of Medicine, Inonu University, Malatya 44280, Turkey; yilmaz.ercan@inonu.edu.tr (E.Y.);; 2 Department of Pediatric, Faculty of Medicine, Inonu University, Malatya 44280, Turkey; 3 Department of Neonatology, Faculty of Medicine, Inonu University, Malatya 44280, Turkeyramazanozdemir@inonu.edu.tr (R.O.)

**Keywords:** multimorbidity, neonatal, prematurity, perinatal, associated factors, multidomain morbidity, preterm birth

## Abstract

**Highlights:**

**What are the main findings?**

- By 7–8 years of age, 69.8% of children born ≤32 weeks had at least one documented morbidity, and 38.1% had documented morbidities across two or more domains.
- Educational/behavioral morbidity was most common (58.7%), followed by respiratory (42.9%) and neurological/neurodevelopmental morbidity (31.7%).
- Growth-related morbidity was associated with lower birth weight and small-for-gestational-age status, whereas neurological/neurodevelopmental morbidity was associated with chorioamnionitis, neonatal sepsis, and longer respiratory support.

**What are the implications of the main findings?**

- Children born very preterm may carry a substantial multidomain morbidity burden into school age.
- Long-term follow-up should extend beyond major neurological sequelae and include educational, behavioral, respiratory, sensory, and growth outcomes.

**Abstract:**

Objective: Despite improved neonatal survival among very preterm infants, health problems affecting various organ systems may persist in the long term. The aim of this study was to determine the burden of morbidity and multimorbidity documented by 7–8 years of age among children born at ≤32 weeks’ gestation and to investigate the perinatal and neonatal factors associated with these outcomes. Methods: A single-center observational cohort study included 63 children born at ≤32 weeks’ gestation and assessed at 7–8 years of age. Perinatal and neonatal data were obtained retrospectively from medical records. Long-term health outcomes were assessed across five morbidity domains: respiratory, neurological/neurodevelopmental, sensory, growth-related, and educational/behavioral. The co-occurrence of two or more morbidity domains was defined as multimorbidity. Results: The median gestational age was 30 weeks (Q1–Q3, 28–32 weeks), and the birth weight was 1.23 kg (Q1–Q3, 0.9–1.65 kg). At least one morbidity was identified in 69.8% of the children, whilst 38.1% had morbidity involving two or more domains. The most common comorbidities were educational/behavioral (58.7%) and respiratory (42.9%). Neurological/neurodevelopmental morbidity was 31.7%, whilst sensory and growth-related morbidities accounted for 28.6%. Growth-related morbidity was associated with lower birth weight (*p* = 0.006) and being small for gestational age (SGA) (*p* < 0.001). Neurological/neurodevelopmental morbidity was associated with chorioamnionitis (*p* = 0.03), longer duration of CPAP (*p* = 0.04) and oxygen therapy (*p* = 0.01), as well as neonatal sepsis (*p* = 0.03). Conclusions: In this cohort, morbidities documented by 7–8 years of age occurred across multiple domains, with conditions in two or more domains co-occurring in a considerable proportion of children born at ≤32 weeks’ gestation. These findings support multidimensional long-term follow-up, while recognizing the heterogeneity of the 25–32-week gestational-age range.

## 1. Introduction

Despite significant advances in neonatal care, preterm birth remains one of the leading causes of childhood mortality and long-term morbidity. In particular, the marked increase in survival rates among very preterm infants born before 32 weeks’ gestation has led to a shift in clinical focus from neonatal survival alone towards long-term functional outcomes. Now that a significant proportion of these children have reached school age, it is increasingly recognized that the effects of prematurity are not limited to the neonatal period; they can persist across a range of interrelated areas, including respiratory function, neurological and neurodevelopmental function, growth, sensory function, behavior and academic performance [1,2].

Among the long-term consequences of preterm birth, neurodevelopmental morbidities are one of the most extensively studied areas. Whilst major neurological sequelae include cerebral palsy, motor impairments and sensory losses, milder but functionally significant cognitive, language, attention, executive function and learning difficulties may also become apparent by school age [1,2,3]. Consequently, a normal or near-normal neurodevelopmental assessment during early childhood does not entirely rule out difficulties that may arise in subsequent school years. Recent systematic reviews indicate that school-aged children born preterm remain at increased risk of difficulties in motor and cognitive functioning, with potential consequences for learning and academic performance [3,4]. In particular, the fact that differences in mathematical performance may persist throughout primary school suggests that the educational consequences of prematurity may be long-term [5].

The respiratory system is also one of the main organ systems affected in the long term by preterm birth. Preterm birth, which occurs during a critical period of lung development, results in immature airways and lung parenchyma being exposed to postnatal factors such as oxygen, mechanical ventilation, inflammation and infection. Whilst bronchopulmonary dysplasia is the best-known clinical phenotype of this process, long-term respiratory morbidity is not limited to children with a history of bronchopulmonary dysplasia. Recurrent respiratory symptoms, wheezing, asthma-like clinical presentations and impaired respiratory function have been reported in preterm children of school age [6,7]. Consequently, when assessing respiratory health following prematurity, it is necessary to look beyond neonatal diagnoses and also take into account clinical outcomes in school-age children.

The effects of prematurity are also significant in terms of growth and sensory functions. In particular, the combination of prematurity with fetal growth restriction or low birth weight for gestational age may pose an additional risk that could adversely affect functional outcomes during school age [8]. Vision and hearing problems should be considered not merely as independent morbidities, but as factors that may affect a child’s language development, learning, academic achievement and social adjustment. Thus, when relatively mild impairments in different organ systems occur together in the same child, they may create a cumulative functional burden that goes beyond the effects of the individual morbidities.

Assessing the long-term outcomes of children born very preterm solely through a single disease or organ system may provide an incomplete picture of their health burden. Previous studies have begun to address this issue from a multidimensional perspective. Neurodevelopmental and behavioral difficulties have been shown to cluster within the same individuals, supporting the concept of heterogeneous multidomain outcome profiles after very preterm birth [9]. In addition, large population-based registry data have demonstrated an increased risk of multimorbidity among individuals born preterm during adolescence [10]. Nevertheless, much of the existing literature continues to examine individual outcomes, such as cerebral palsy, bronchopulmonary dysplasia, cognitive impairment, or academic performance, separately. Data remain limited on the simultaneous assessment of respiratory, neurological/neurodevelopmental, sensory, growth-related, and educational/behavioral morbidities within the same school-age cohort and on how perinatal and neonatal factors relate to these different morbidity domains.

It is thought that not only gestational age and birth weight but also various exposures during the intrauterine and neonatal periods may play a role in predicting long-term morbidity. Inflammatory and intensive care related factors, such as chorioamnionitis, neonatal sepsis, mechanical ventilation and prolonged respiratory support, may have lasting effects on the developing brain and other organ systems. In particular, neonatal sepsis has been reported to be associated with a higher risk of neurodevelopmental disorders in very preterm infants [11]. Similarly, whilst the relationship between chorioamnionitis and long-term neurodevelopment has been investigated, the results are heterogeneous, and the independent effect of this association has not yet been fully elucidated [12,13]. Recent systematic reviews also emphasize that it remains difficult to predict neurodevelopmental outcomes in very preterm infants at later ages during the early stages, and that the relationship between neonatal risk factors and long-term outcomes needs to be better defined [14].

This study aimed to assess the health burden at 7–8 years of age among children born at ≤32 weeks’ gestation using a multidimensional approach that considers the areas of respiratory, neurological/neurodevelopmental, sensory, growth-related and educational/behavioral morbidity collectively. Furthermore, the study aimed to determine the burden of morbidities both individually and in combination across different domains, and to investigate the associations between long-term morbidity domains and maternal, perinatal and neonatal characteristics.

## 2. Materials and Methods

### 2.1. Study Design and Participants

This single-center observational cohort study was designed to evaluate the long-term health outcomes of school-aged children born at ≤32 weeks’ gestation and to identify the perinatal and neonatal factors associated with these outcomes. The study included children who had been followed up in the Neonatal Intensive Care Unit of a tertiary teaching and research hospital and who had reached the age of 7–8 years at the time of the study. The neonatal intensive care unit is a tertiary-level referral unit providing comprehensive care for very preterm and critically ill neonates, including invasive and non-invasive respiratory support, oxygen therapy, surfactant administration, and management of major complications of prematurity. Home oxygen requirement at discharge was also recorded from neonatal medical records. During the birth period of the study cohort, approximately 7–8 years before the present assessment, the unit provided the same overall level of neonatal intensive care and the principal respiratory and supportive treatments were available. Although routine clinical protocols and equipment have evolved over time, there was no major change in the level or scope of intensive care that would substantially alter the interpretation of neonatal exposures in this cohort. Following approval from the ethics committee, potentially eligible patients were identified using the neonatal intensive care unit’s electronic record system. Parents were contacted by telephone using the contact details on file, and those who agreed to participate were invited to the Pediatric Allergy Outpatient Clinic for assessment. The Pediatric Allergy Outpatient Clinic was used solely as the location for study assessments because it provided an appropriate and readily available outpatient setting; participants were not selected or referred on the basis of allergic or respiratory symptoms or diagnoses. All assessments were completed during a single outpatient clinic visit on the same day.

The inclusion criteria for the study were defined as: (1) Gestational age of ≤32 weeks. There was no fixed lower gestational-age threshold for provision of active neonatal care; decisions were based on the infant’s gestational age, clinical condition, and expected viability. In the present cohort, the lowest gestational age among survivors included in the study was 25 weeks. In addition, the criteria were (2) availability of medical records from the neonatal period, (3) being aged 7–8 years at the time of the study, and (4) written informed consent from a parent or legal guardian for participation in the study. Children with incomplete data or whose families did not give consent were excluded from the study.

**Figure 1:**
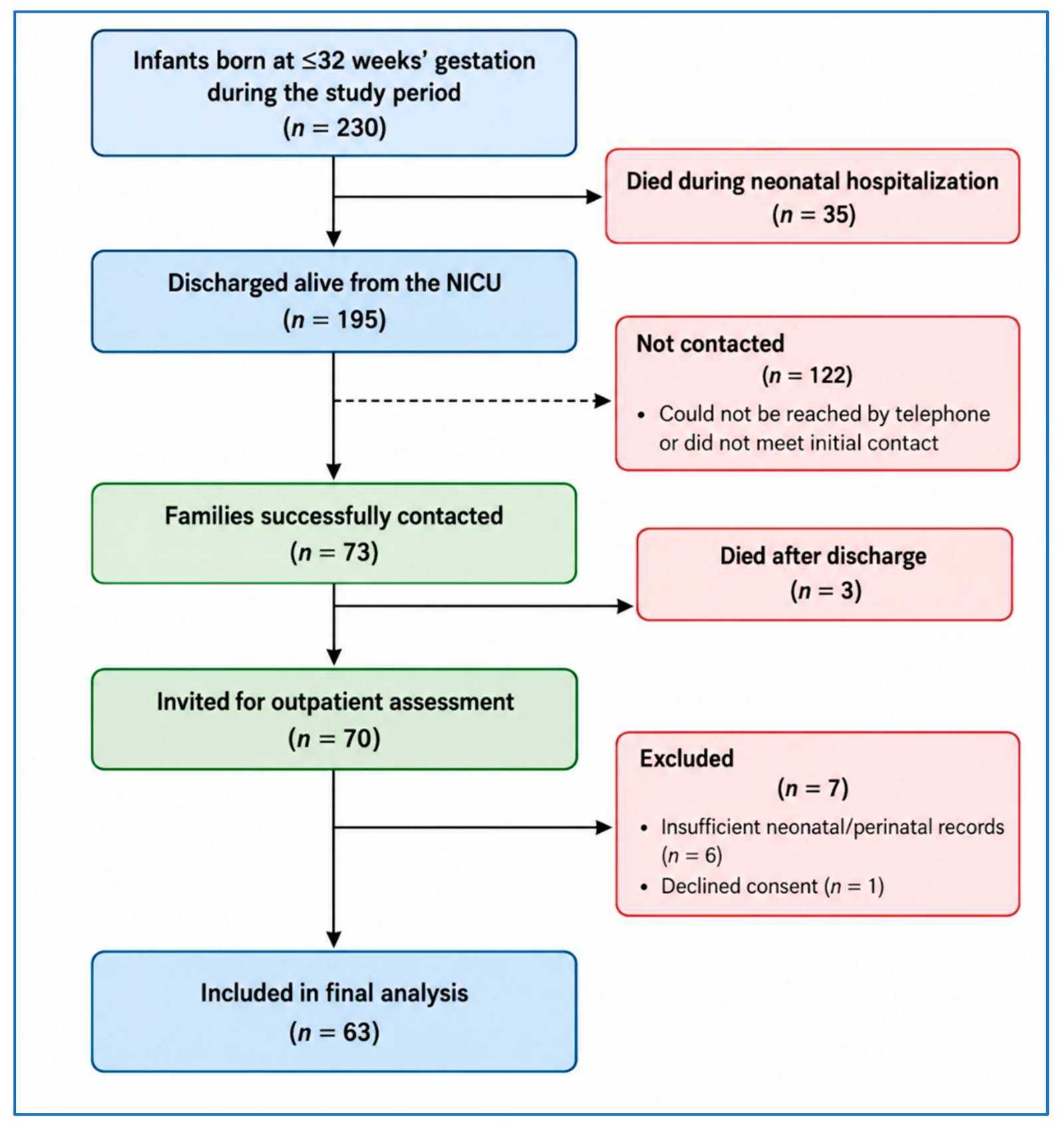
Flow chart of participant selection and study enrollment.

The study cohort comprised children born at ≤32 weeks’ gestation who were 7–8 years of age at the time of assessment. Overall, 230 infants meeting the gestational-age criterion were born at our center between July 2017 and July 2019; 35 died during the neonatal hospitalization, and 195 were discharged alive from the NICU. Following ethics approval on 30 June 2026, recruitment and outpatient assessments were conducted between 1 and 20 July 2026. Seventy-three families were successfully contacted and agreed to participate, and these children were invited for clinical assessment. Three children were subsequently found to have died after NICU discharge and were therefore excluded. Of the remaining 70 children, seven were excluded—six because of insufficient neonatal or perinatal records and one because consent was ultimately declined—resulting in 63 children in the final analysis (Figure 1).

### 2.2. Collection of Perinatal and Neonatal Data

Perinatal and neonatal data were obtained retrospectively from hospital records and neonatal intensive care unit files. Maternal/perinatal variables recorded included mode of delivery, gestational age, birth weight, small for gestational age (SGA), maternal smoking during pregnancy, antenatal corticosteroid exposure, maternal pre-eclampsia and chorioamnionitis. In addition, a history of breastfeeding for more than six months was assessed. Breastfeeding for more than six months was defined as receiving any breast milk for >6 months, irrespective of concurrent formula or complementary feeding. Variables relating to the neonatal period included the need for and duration of mechanical ventilation, continuous positive airway pressure (CPAP) treatment and its duration, duration of oxygen therapy, length of stay in the neonatal intensive care unit (NICU) and surfactant treatment. Neonatal morbidities assessed included patent ductus arteriosus (PDA), neonatal sepsis, necrotizing enterocolitis (NEC), intraventricular hemorrhage (IVH), neonatal apnea, retinopathy of prematurity (ROP) and bronchopulmonary dysplasia (BPD). PDA cases were classified as either hemodynamically stable and not requiring specific treatment or clinically significant and requiring treatment. NEC was staged according to the modified Bell criteria, and IVH according to the Papile grading system. ROP was recorded as clinically significant only when treatment was required. BPD was defined as the requirement for supplemental oxygen at 36 weeks’ postmenstrual age, while severe ROP was defined as ROP requiring treatment. For ROP, only cases requiring treatment were considered clinically significant and recorded as neonatal morbidity, whereas infants with ROP not requiring treatment were not classified as having this morbidity in the present study. Neonatal apnea was defined as any documented apnea episode during NICU hospitalization, without etiological subclassification because the retrospective records did not reliably distinguish apnea of prematurity from secondary causes.

### 2.3. Assessment and Identification of Long-Term Morbidities

Long-term health outcomes at 7–8 years of age were determined using clinical assessment, parental interviews and existing medical records. Morbidities were classified into five main areas: respiratory, neurological/neurodevelopmental, sensory, growth-related and educational/behavioral. No uniform severity threshold was applied across morbidity domains because the included conditions were heterogeneous; therefore, the domain-based classification reflects the presence of documented conditions rather than their severity or functional impact. Morbidities were identified using predefined domain-specific criteria based on clinical examination, documented physician or specialist diagnoses, medical records, school/teacher assessments, and standardized anthropometric measurements, as appropriate for each outcome. The presence of at least one identified health problem in any area was considered to indicate the presence of morbidity in that area. The co-occurrence of two or more different areas of morbidity in a single child was defined as multimorbidity. Accordingly, these outcomes represent conditions documented by the time of the 7–8-year assessment and were not necessarily newly diagnosed or uniformly reassessed at that visit.

Respiratory morbidities: Respiratory morbidities included chronic structural lung disease, a history of adenoid hypertrophy, asthma, allergic rhinitis, pneumonia requiring admission to intensive care within the first two years of life, and recurrent lower respiratory tract infections. Chronic structural lung disease was defined as a physician-documented persistent structural pulmonary abnormality beyond the neonatal period. In the present cohort, this category included bronchiectasis in one child and persistent fibrotic changes on thoracic imaging in another. This long-term morbidity was considered distinct from neonatal bronchopulmonary dysplasia (BPD). Recurrent lower respiratory tract infection was defined as recurrent physician-diagnosed pneumonia or two or more episodes of bronchiolitis. Asthma and allergic rhinitis were identified based on previously established physician diagnoses documented in the medical records and were confirmed by review of the diagnostic history and current clinical assessment during the study visit. Viral-induced wheeze without an established diagnosis of asthma was not classified as asthma. Adenoid hypertrophy was defined as a previously documented diagnosis made by a pediatrician or otorhinolaryngologist. Data on the degree of upper-airway obstruction, obstructive sleep apnea, or need for surgical intervention were not systematically available. The presence of at least one of these conditions was considered to constitute respiratory morbidity.

Sensory morbidities: Sensory morbidities encompassed disorders related to vision and hearing. Vision-related morbidity was defined as the presence of a visual impairment that had previously been assessed by an ophthalmologist and documented in medical records. The use of corrective spectacles due to a refractive error was also considered within the scope of vision-related morbidity. Hearing-related morbidity, on the other hand, was defined as the presence of hearing loss that had been diagnosed following an audiological assessment and/or an assessment by an ear, nose and throat specialist and documented in medical records. The presence of at least one of these findings was classified as sensory morbidity.

Educational and behavioral morbidities: This domain included special educational needs, moderate-to-poor mathematics achievement, attention-deficit/hyperactivity disorder (ADHD), sleep disturbances, and school adjustment problems. Mathematics performance was classified as good, moderate, or poor based on the most recent school report and current teacher evaluation; no standardized academic achievement test was administered. Moderate or poor mathematics performance was considered an educational morbidity. ADHD was defined by a previously established physician diagnosis documented in the medical records; no additional standardized ADHD assessment was performed during the study visit. Special educational needs were identified from a current special educational needs report and/or receipt of special educational support. Sleep disturbances and school adjustment problems were recorded based on parental history and, where available, supported by school or teacher assessments.

Neurological and neurodevelopmental conditions: This category included epilepsy, cerebral palsy, motor developmental delay, speech and language delay, and learning difficulties. Conditions were identified from previously documented physician diagnoses, developmental assessments, medical records, and, where available, relevant specialist evaluations. No standardized neurodevelopmental reassessment was performed during the study visit. The presence of at least one condition was classified as neurological/neurodevelopmental morbidity.

Growth-Related Morbidities: Height and weight were measured using standardized methods during the study clinic visit, and age- and sex-specific height and weight standard deviation scores (SDSs) were calculated according to national reference data [15]. Short stature was defined as a height SDS < −2, and low body weight was defined as a weight SDS < −2. The presence of either short stature or low body weight was considered a growth-related morbidity.

### 2.4. Statistical Analysis

Statistical analyses were performed using IBM SPSS Statistics for Windows, version 25.0, IBM Corp., Armonk, NY, USA. The normality of continuous variables was assessed using visual methods and the Shapiro–Wilk test. Non-normally distributed variables were presented as median (Q1–Q3). Categorical variables were expressed as counts and percentages. The associations between perinatal and neonatal factors and long-term morbidity domains were analyzed separately for each morbidity domain. The Pearson chi-square test was used to compare categorical variables; where the expected cell count was low, Fisher’s exact test was used. The Mann–Whitney U test was used to compare continuous variables between two independent groups. Analyses were carried out separately for respiratory, neurological/neurodevelopmental, sensory, educational/behavioral and growth-related morbidities. All tests were assessed as two-tailed, and *p* < 0.05 was considered statistically significant.

## 3. Results

### 3.1. Perinatal and Neonatal Characteristics

Of the 63 children included in the study, 26 (41.3%) were male. The median gestational age was 30 weeks (Q1–Q3, 28–32 weeks), and the median birth weight was 1.23 kg (Q1–Q3, 0.9–1.65 kg). Fifteen children (23.8%) were small for gestational age. Maternal smoking during pregnancy was reported in 14 (22.2%) cases, while maternal preeclampsia and chorioamnionitis were present in 16 (25.4%) and 11 (17.5%) pregnancies, respectively. Antenatal corticosteroid exposure was documented in 21 (33.3%) children.

Regarding neonatal respiratory support, 23 (36.5%) children required mechanical ventilation and 57 (90.5%) received CPAP treatment. Mechanical ventilation and CPAP were not mutually exclusive; all 23 infants who received mechanical ventilation subsequently received CPAP, and the 57 infants receiving CPAP included both post-extubation and primary CPAP use. The median durations of mechanical ventilation, CPAP treatment, and oxygen therapy were 0 days (Q1–Q3, 0–1 day), 9 days (Q1–Q3, 4–21 days), and 13 days (Q1–Q3, 8–24 days), respectively. Three infants (4.8%) were discharged home on supplemental oxygen. The median NICU length of stay was 45 days (Q1–Q3, 29–75 days), and 12 (19.0%) children received surfactant treatment. Neonatal sepsis was observed in 37 (58.7%) children, while bronchopulmonary dysplasia occurred in 19 (30.2%).

Other neonatal morbidities included PDA in 15 infants (23.8%); eight had hemodynamically stable PDA that did not require specific treatment, whereas seven required treatment. IVH was documented in 12 infants (19.0%), including grade 1 IVH in 11 and grade 2 IVH in one; no grade 3 or 4 IVH was observed. NEC occurred in two infants (3.2%), with one case classified as stage IA and the other as stage IIA. ROP requiring treatment was documented in three infants (4.8%). The perinatal and neonatal characteristics of the study population are presented in Table 1.

### 3.2. Morbidity and Multimorbidity Documented by 7–8 Years of Age

By 7–8 years of age, 44 children (69.8%) had at least one documented morbidity, while 24 (38.1%) had morbidities documented across two or more domains. Educational and behavioral morbidities were the most prevalent domain, documented in 37 (58.7%) children, followed by respiratory morbidity in 27 (42.9%), neurological and neurodevelopmental morbidity in 20 (31.7%), and sensory and growth-related morbidity, each documented 18 (28.6%) children.

Among respiratory outcomes, a history of adenoid hypertrophy was documented in 15 (23.8%) children, allergic rhinitis in 12 (19.0%), asthma in 7 (11.1%), and recurrent lower respiratory tract infections in 5 (7.9%). Chronic structural lung disease and pneumonia requiring intensive care admission within the first 2 years of life were each reported in 2 (3.2%) children. Within the neurological and neurodevelopmental domain, speech and language delay were documented in 18 (28.6%) children, motor developmental delay in 12 (19.0%), learning difficulties in 9 (14.3%), cerebral palsy in 3 (4.7%), and epilepsy in 1 (1.6%).

Among sensory outcomes, vision disorders were documented in 16 (25.4%) children and hearing impairment in 3 (4.8%). Growth-related morbidities included underweight in 16 (25.4%) children and short stature in 7 (11.1%). Regarding educational and behavioral outcomes, moderate-to-poor mathematics performance was documented in 17 (27.0%) children, attention-deficit/hyperactivity disorder in 13 (20.6%), requirement for special education in 7 (11.1%), restless sleep in 7 (11.1%), and school adjustment difficulties in 5 (7.9%). Health outcomes and the multimorbidity burden documented by 7–8 years of age are summarized in Table 2.

Additionally, regarding educational and rehabilitative support, 7 (11.1%) children received support from a special education center, while 4 (6.3%) had an Individualized Education Program (IEP). Physical therapy was provided to 10 (15.9%) children, speech therapy to 7 (11.1%), and occupational therapy to 2 (3.2%).

### 3.3. Associations Between Perinatal and Neonatal Factors and Morbidity Domains

In exploratory analyses, growth-related morbidity was associated with lower birth weight [median 0.97 kg (Q1–Q3, 0.79–1.25); *p* = 0.006] and small-for-gestational-age status [12 (66.7%); *p* < 0.001]. Chorioamnionitis was associated with neurological and neurodevelopmental morbidity [8 (40.0%); *p* = 0.03]. Children with neurological and neurodevelopmental morbidity also had a longer duration of CPAP treatment [median 16 days (Q1–Q3, 7–35 days); *p* = 0.04], a longer duration of oxygen therapy [median 14 days (Q1–Q3, 10–45 days); *p* = 0.01], and a higher frequency of neonatal sepsis [16 (80.0%); *p* = 0.03]. Although they did not meet the predefined threshold for statistical significance, several additional associations were observed at *p* = 0.05. Maternal preeclampsia was more frequent among children with sensory morbidity [8 (44.4%); *p* = 0.05] and growth-related morbidity [8 (44.4%); *p* = 0.05]. In addition, children with growth-related morbidity had a longer duration of oxygen therapy [median 16 days (Q1–Q3, 12–35 days); *p* = 0.05]. No statistically significant associations were identified between the evaluated perinatal or neonatal factors and respiratory or educational and behavioral morbidity domains. The associations between perinatal and neonatal factors and morbidity domains at the age of 7–8 years are presented in Table 3.

## 4. Discussion

In this study, the long-term health outcomes at 7–8 years of age for children born at ≤32 weeks’ gestation were assessed using a multidimensional approach, and it was demonstrated that a significant proportion of these children continued to bear a burden of morbidity during their school years. The main findings were that, by 7–8 years of age, more than two-thirds of the children had at least one documented morbidity and more than one-third had morbidities across two or more domains. Educational and behavioral morbidity was the most frequently documented domain, followed by respiratory and neurological/neurodevelopmental morbidity. Exploratory analyses showed nominal associations of low birth weight and small-for-gestational-age status with growth-related morbidity, and of chorioamnionitis, prolonged CPAP and oxygen therapy, and neonatal sepsis with neurological/neurodevelopmental morbidity. These findings should be interpreted cautiously and do not establish independent or causal effects. In contrast, no significant association was found between the neonatal factors assessed—including gestational age, mechanical ventilation and bronchopulmonary dysplasia (BPD)—and respiratory or educational/behavioral morbidities. These findings support assessing the long-term effects of very preterm birth not solely in terms of a single organ system or major disability, but also by considering the co-occurrence of documented morbidities across different health domains.

By 7–8 years of age, 69.8% of children had at least one documented morbidity, and 38.1% had morbidities across two or more domains. These findings describe the proportion of children with documented conditions and the co-occurrence of conditions across morbidity domains; they should not be interpreted as measures of clinical severity or functional impact. Long-term studies show that very preterm infants may experience difficulties in motor, cognitive, behavioral, educational and sensory domains even in the absence of major sequelae such as cerebral palsy or severe cognitive impairment. Synnes and Hicks emphasized that neurodevelopmental effects following preterm birth can persist into school-age and adulthood, and that criteria for severe disability alone do not adequately reflect the long-term burden [1]. More recently, a study analyzing extensive Finnish and Norwegian registries also showed that preterm birth is associated with an increased risk of multimorbidity—characterized by the co-occurrence of two or more health problems during adolescence [10]. In this regard, our findings suggest that multimorbidity may be clinically significant even at an earlier stage following preterm birth, namely during school-age.

It is particularly significant that educational and behavioral morbidities were the most frequently documented domain (58.7%). Moderate-to-poor maths achievement was observed in 27.0% of children, whilst attention deficit/hyperactivity disorder (ADHD) was identified in 20.6%. A systematic review evaluating school-age outcomes following preterm birth reported that children born preterm are at a higher risk of impairments in motor development, behavior and school performance compared with their term peers [3]. Similarly, in a longitudinal study in which very preterm children were followed throughout primary school, it was shown that their arithmetic performance was on average 0.53 standard deviations lower than that of their term-born peers, and that this difference persisted throughout their school years [5]. The high prevalence of difficulties in maths performance in our study is consistent with this literature. Furthermore, the prevalence of ADHD at 20.6% is also noteworthy. A meta-analysis by Franz and colleagues demonstrated that the likelihood of an ADHD diagnosis in very preterm or very low birth weight children was approximately three times higher than in term or normal birth weight controls, with the risk increasing further as the degree of prematurity increased [16]. There is also evidence suggesting that the impact of prematurity on executive functions may contribute to behavioral problems [17]. Taken together, these findings suggest that follow-up of very preterm children should extend beyond neurological examination and growth assessment to include academic performance, attention, behavior, and school adjustment.

Neurological and neurodevelopmental morbidities were documented in 31.7% of the children by 7–8 years of age; the most common findings were speech and language delay (28.6%) and motor development delay (19.0%). In contrast, cerebral palsy was found in 4.7% of cases, whilst epilepsy was present in 1.6%. This distribution is significant in that it demonstrates that the long-term neurodevelopmental burden of preterm birth today does not solely comprise major disorders such as cerebral palsy. Although major neurological sequelae are observed at relatively low rates, milder problems in the areas of language, motor function, learning and behavior may become apparent during school age [1,3]. For this reason, monitoring during school age may be critical in identifying neurodevelopmental difficulties that were not detected in early childhood or that become more apparent as academic demands increase.

One of our most striking findings in terms of neurodevelopmental morbidity is the association with neonatal sepsis. The fact that 80% of children with neurological/neurodevelopmental morbidity had a history of neonatal sepsis, and that this association was statistically significant, is consistent with the potential long-term effects of early postnatal inflammation on the developing brain. A systematic review and meta-analysis examining neonatal sepsis and neurodevelopmental outcomes in very preterm infants reported that the risk of neurodevelopmental disorders—including cerebral palsy and neurosensory impairments—was significantly higher in infants who had experienced sepsis [11]. It is thought that systemic inflammation, the cerebral inflammatory response, hemodynamic instability and associated intensive care exposure may underlie this association. However, due to the observational nature of our study and the sample size, it is not possible to establish the independent causal effect of neonatal sepsis.

Similarly, maternal chorioamnionitis has been found to be associated with neurological/neurodevelopmental morbidity. It has long been debated that intrauterine exposure to chorioamnionitis may affect the developing central nervous system via a fetal inflammatory response. A large cohort study in Sweden, covering over two million births, demonstrated that exposure to chorioamnionitis is associated with risks of cerebral palsy, autism, ADHD and intellectual disability; however, it was reported that a significant proportion of this association was mediated by preterm birth [18]. Consequently, whilst the association between chorioamnionitis and neurodevelopment in our study is biologically meaningful, it should be interpreted in light of the potential confounding effects of gestational age and other neonatal factors.

It is also noteworthy that children with neurological/neurodevelopmental morbidity require longer periods of CPAP and oxygen therapy. Rather than indicating that respiratory support directly causes neurodevelopmental damage, this finding should be interpreted as an indicator of the severity of neonatal illness. It is likely that more immature and clinically more severely affected newborns require respiratory support for longer periods and are also more exposed to inflammation, hypoxemia, infection and other neonatal complications. Therefore, the duration of CPAP and oxygen therapy may serve as readily available clinical markers of long-term neurodevelopmental risk; however, our current data do not permit the establishment of causality.

In our study, respiratory morbidities were documented in 42.9% of the children by 7–8 years of age; adenoid hypertrophy (23.8%), allergic rhinitis (19.0%), and asthma (11.1%) were the most frequently reported conditions. As preterm birth occurs during a critical period of lung development, and the developing airways are exposed to oxygen, mechanical ventilation and inflammation, this may affect long-term respiratory health. It has been reported that airway obstruction and bronchial lability may be observed in school-aged preterm children, regardless of the presence of BPD [19]. It has also been demonstrated that respiratory function may continue to be adversely affected during school age in preterm children with a history of BPD [20]. However, in our study, no significant association was found between BPD, mechanical ventilation, duration of oxygen therapy or gestational age and clinical respiratory morbidity. This finding, alongside the limited sample size, suggests that the respiratory phenotype in school-aged children may not be explained solely by neonatal lung damage. Postnatal environmental exposures, infections, atopic predisposition and individual differences in airway development may influence long-term respiratory outcomes.

Sensory impairments were documented in 28.6% of the children by 7–8 years of age, with visual impairments accounting for the majority (25.4%). The impact of very preterm birth on visual outcomes in school-age children is well established. A study evaluating seven-year-old children born at ≤32 weeks reported that, compared with term controls, preterm children were more likely to require spectacles, have reduced visual acuity, suffer from impaired stereopsis and have strabismus [21]. Large-scale national registry studies have also demonstrated that very preterm birth carries an increased risk of visual and hearing impairments [22]. Although a borderline significant association was found in our study between maternal pre-eclampsia and sensory morbidity (*p* = 0.05), this finding should be interpreted with caution due to the sample size and multiple comparisons.

Growth-related morbidity was documented in 28.6% of the children by 7–8 years of age, and low birth weight and SGA were strongly associated with this outcome. It is particularly noteworthy that 66.7% of children with growth-related morbidity were SGA. Studies evaluating the long-term outcomes of preterm and SGA infants have shown that intrauterine growth restriction may affect not only postnatal growth but also neuromotor, cognitive and educational outcomes [23]. Our findings support the notion that the pattern of fetal growth may leave a lasting impact on physical growth during school age and highlight the importance of long-term anthropometric follow-up in preterm-SGA children.

This study has several limitations. First, its single-center design and limited sample size reduced statistical power, particularly for uncommon morbidities, and precluded adequately powered subgroup analyses across narrower gestational-age categories. Given the biological and clinical heterogeneity of the 25–32-week gestational-age range, residual heterogeneity may therefore have influenced the observed associations. Second, the large number of exploratory comparisons across perinatal and neonatal factors increased the risk of type I error. Because no formal adjustment for multiple testing was applied, nominal *p* values and unadjusted associations should be interpreted cautiously and considered hypothesis-generating rather than causal. Third, the absence of a term-born control group prevents direct comparison of morbidity rates with those of children born at term. In addition, some long-term outcomes relied on retrospective medical records, parental reports, or previously documented clinical assessments, which may have introduced heterogeneity or misclassification. Furthermore, the timing of historical diagnoses and school-based assessments was not uniformly available; therefore, some morbidities may have been identified before the 7–8-year study assessment rather than diagnosed contemporaneously at that age. In particular, academic performance was not evaluated using standardized achievement testing, neurological/neurodevelopmental outcomes were not reassessed using a uniform standardized instrument at 7–8 years, and the severity or functional impact of some respiratory diagnoses, especially adenoid hypertrophy, was not systematically characterized. Moreover, the binary morbidity classification did not account for differences in severity or functional impact; consequently, conditions within the same domain may have substantially different implications for the child’s daily functioning and family burden.

In addition, exclusion of children with incomplete neonatal/perinatal records may have introduced selection bias, and the small number of infants discharged on supplemental oxygen prevented separate evaluation of this marker of neonatal respiratory severity. Only 63 of the 195 children discharged alive from the NICU were included in the school-age assessment. Participating children may have differed from those who could not be contacted, and this nonresponse may have affected the observed morbidity frequencies and limited their generalizability. Nevertheless, the assessment of multiple health domains within the same school-age cohort and the availability of detailed neonatal data are important strengths of the study.

## 5. Conclusions

Our findings indicate that, by 7–8 years of age, children born at ≤32 weeks’ gestation had documented morbidities across multiple health domains, with morbidities in more than one domain co-occurring in a considerable proportion of children. In particular, the high proportion of children with documented educational and behavioral problems, together with the co-occurrence of morbidities across different domains, suggests that follow-up focused solely on major neurological or respiratory sequelae may be insufficient. The observed associations of SGA and low birth weight with growth-related morbidity, and of chorioamnionitis, neonatal sepsis, and prolonged respiratory support with neurological/neurodevelopmental morbidity, should be considered exploratory. These neonatal factors warrant further investigation and require confirmation in larger prospective cohorts before their potential role in long-term risk stratification can be determined. These findings support the importance of multidisciplinary and multidimensional follow-up of very preterm infants until school age.

## Figures and Tables

**Table 1:** Perinatal and neonatal characteristics of the study population.

Variables	n (%)
Male sex	26 (41.3)
Cesarean delivery	61 (96.8)
Gestational age, weeks, median (Q1–Q3)	30 (28–32)
Birth weight, kg, median (Q1–Q3)	1.23 (0.9–1.65)
Small for gestational age	15 (23.8)
Maternal smoking during pregnancy	14 (22.2)
Antenatal corticosteroid exposure	21 (33.3)
Breastfeeding for >6 months	30 (47.6)
Maternal preeclampsia	16 (25.4)
Chorioamnionitis	11 (17.5)
Mechanical ventilation	23 (36.5)
Duration of mechanical ventilation, days, median (Q1–Q3)	0 (0–1)
CPAP treatment	57 (90.5)
Duration of CPAP treatment, days, median (Q1–Q3)	9 (4–21)
Duration of oxygen therapy, days, median (Q1–Q3)	13 (8–24)
NICU length of stay, days, median (Q1–Q3)	45 (29–75)
Surfactant treatment	12 (19)
Neonatal PDA	15 (23.8%)
Neonatal sepsis	37 (58.7)
NEC	2 (3.2)
IVH	12 (19)
Neonatal apnea	23 (36.5)
ROP requiring treatment	3 (4.8)
Bronchopulmonary dysplasia	19 (30.2)

**Table 2:** Morbidities documented by 7–8 years of age and multimorbidity burden.

Morbidities	n (%)
Respiratory Morbidities	27 (42.9)
Chronic structural lung disease	2 (3.2)
History of adenoid hypertrophy	15 (23.8)
Asthma	7 (11.1)
Allergic rhinitis	12 (19)
Pneumonia requiring intensive care admission within the first 2 years of life	2 (3.2)
Recurrent lower respiratory tract infection	5 (7.9)
Neurological and Neurodevelopmental Morbidities	20 (31.7)
Epilepsy	1(1.6)
Cerebral palsy	3 (4.7)
Motor developmental delay	12 (19)
Speech and language delay	18 (28.6)
Learning difficulties	9 (14.3)
Sensory Morbidities	18 (28.6)
Vision disorder	16 (25.4)
Hearing impairment	3 (4.8)
Growth-related Morbidity	18 (28.6)
Short stature (height SDS < −2)	7 (11.1)
Underweight (weight SDS < −2)	16 (25.4)
Educational and Behavioral Morbidities	37 (58.7)
Requirement for special education	7 (11.1)
Moderate-to-poor mathematics performance	17 (27)
Attention-deficit/hyperactivity disorder	13 (20.6)
Restless sleep	7 (11.1)
School adjustment difficulties	5 (7.9)
≥2 morbidity domains	24 (38.1)
Total morbidity	44 (69.8)

**Table 3:** Associations between perinatal and neonatal factors and morbidity domains documented by 7–8 years of age.

Variable	Respiratory Morbidities (n = 27)		Neurological and Neurodevelopmental Morbidities (n = 20)		Sensory Morbidities (n = 18)		Educational and Behavioral Morbidities (n = 37)		Growth-Related Morbidity (n = 18)	
	n (%)	*p*-Value	n (%)	*p*-Value	n (%)	*p*-Value	n (%)	*p*-Value	n (%)	*p*-Value
Male sex	11 (40.7)	1.0	11 (55)	0.21	7 (38.9)	1.0	14 (37.8)	0.68	6 (33.3)	0.59
Gestational age, weeks, median (Q1–Q3)	29 (27–32)	0.33	29 (27–31)	0.27	29 (26–32)	0.76	30 (26–32)	0.95	29 (27–32)	0.54
Birth weight, kg, median (Q1–Q3)	1.2 (0.9–1.65)	0.95	1.04 (0.89–1.42)	0.12	1.2 (0.87–2.0)	0.74	1.25 (0.9–1.69)	0.51	0.97 (0.79–1.25)	0.006
Small for gestational age	6 (22.2)	1.0	7 (35)	0.20	6 (33.3)	0.33	7 (18.9)	0.41	12 (66.7)	<0.001
Antenatal corticosteroid exposure	10 (37)	0.78	5 (25)	0.50	5 (27.8)	0.76	13 (35.1)	0.92	4 (22.2)	0.37
Maternal smoking during pregnancy	6 (22.2)	1.0	6 (30)	0.34	4 (22.2)	1.0	7 (18.9)	0.65	2 (11.1)	0.31
Maternal pre-eclampsia	6 (22.2)	0.83	5 (25)	1.0	8 (44.4)	0.05	10 (27)	0.95	8 (44.4)	0.05
Chorioamnionitis	5 (18.5)	1.0	8 (40)	0.03	6 (33.3)	0.06	7 (18.9)	1.0	3 (16.7)	1.0
Mechanical ventilation	8 (29.6)	0.47	10 (50)	0.21	6 (33.3)	0.96	15 (40.5)	0.59	8 (44.4)	0.59
Duration of mechanical ventilation, days, median (Q1–Q3)	0 (0–1)	0.42	1 (0–3)	0.14	0 (0–2)	0.95	0 (0–2)	0.26	0 (0–4)	0.23
Surfactant treatment	5 (18.5)	1.0	5 (25)	0.49	5 (27.8)	0.29	8 (21.6)	0.76	4 (22.2)	0.72
CPAP treatment	24 (88.9)	0.64	20 (100)	0.16	17 (94.4)	1.0	35 (94.6)	0.64	17 (94.4)	1.0
Duration of CPAP treatment, days, median (Q1–Q3)	12 (4–26)	0.58	16 (7–35)	0.04	8 (3–26)	0.87	10 (5–23)	0.34	11 (6–29)	0.11
Duration of oxygen therapy, days, median (Q1–Q3)	14 (7–34)	0.85	14 (10–45)	0.01	11 (4–32)	0.31	13 (8–32)	0.78	16 (12–35)	0.05
NICU length of stay, days, median (Q1–Q3)	49 (28–83)	0.87	66 (47–81)	0.87	41 (11–85)	0.97	50 (30–83)	0.39	55 (38–86)	0.17
Neonatal sepsis	15 (55.6)	0.85	16 (80)	0.03	12 (66.7)	0.59	22 (59.5)	1.0	11 (61.1)	1.0
NEC	1 (3.7)	1.0	1 (5)	0.53	1 (5.6)	0.49	2 (5.4)	NC	1 (5.6)	0.49
IVH	6 (22.2)	0.81	3 (15)	0.73	3 (16.7)	1.0	8 (21.6)	0.76	6 (33.3)	0.08
Neonatal apnea	11 (40.7)	0.73	10 (50)	0.21	7 (38.9)	1.0	16 (43.2)	0.29	7 (38.9)	1.0
Bronchopulmonary dysplasia	10 (37)	0.45	9 (45)	0.14	4 (22.2)	0.57	13 (35.1)	0.45	7 (38.9)	0.51

CPAP: Continuous positive airway pressure; IVH: intraventricular hemorrhage; NEC: necrotizing enterocolitis; NICU: neonatal intensive care unit.

## Data Availability

The data presented in this study are available from the corresponding author upon reasonable request because they contain confidential patient information.
